# The association between shorter disease course and sarcopenia in women with endometriosis: a retrospective analysis of NHANES 1999–2006

**DOI:** 10.1038/s41598-025-03511-9

**Published:** 2025-05-25

**Authors:** Litao Sun, Yishu Tian, Lei Ling

**Affiliations:** 1 Center for Reproductive Medicine, Department of Ultrasound Medicine, Zhejiang Provincial People’s Hospital (Affiliated People’s Hospital, Hangzhou Medical College), Hangzhou, China; 2 Center for Reproductive Medicine, Department of Clinical Engineering, Zhejiang Provincial People’s Hospital (Affiliated People’s Hospital, Hangzhou Medical College), Hangzhou, 310014 China

**Keywords:** Disease course, Endometriosis (EMT), Sarcopenia, The national health and nutrition examination survey (NHANES), Metabolic disorders, Nutrition disorders, Reproductive disorders

## Abstract

Endometriosis is a common gynecological disorder that is associated with chronic pelvic pain, infertility, and metabolic complications. Sarcopenia, characterized by progressive skeletal muscle loss, predominantly affects older adults. This study explored the incidence and risk factors for sarcopenia in endometriosis patients using the NHANES dataset, which included 373 participants. Endometriosis was confirmed through self-report questionnaire, and sarcopenia was diagnosed via dual-energy X-ray absorptiometry. Covariates encompassed age, race, marital status, education attainment, poverty income ratio, smoking habits, and comorbidities. Statistical analyses were conducted using SPSS version 26.0, incorporating four multivariate regression models. The average age was 40.3 and 40.0 years in endometriotic participants with and without sarcopenia, respectively. Minority ethnicity had higher odds for sarcopenia (OR 6.00, 95% CI 1.24–29.07). A disease duration of endometriosis less than five years was associated with higher sarcopenia risk (OR 4.83, 95% CI 2.57–9.09). Conversely, lower educational levels were linked to a reduced chance of developing sarcopenia (OR 0.42, 95% CI 0.21–0.86). These findings were consistent across all regression models, indicating that ethnic minority status, higher educational attainment, and shorter disease duration are significant risk factors for concurrent sarcopenia in endometriosis patients.

## Introduction

Endometriosis is a chronic gynecological disorder characterized by the growth of endometrial-like tissue outside the uterine cavity^1^. This ectopic tissue responds to cyclic hormonal changes. It can trigger chronic inflammation, pelvic pain, dysmenorrhea, dyspareunia, and infertility^1^. The exact etiology remains unclear, but proposed theories include retrograde menstruation, coelomic metaplasia, and genetic and immunological factors. Despite affecting approximately 10% of women of reproductive age, endometriosis remains under-researched and underdiagnosed^1^. These gaps highlight the need for enhanced research efforts and increased clinical awareness to improve timely diagnosis and therapeutic interventions. Long-term complications include chronic pelvic pain, subfertility or infertility, bowel and bladder dysfunction, and some metabolic disturbances such as obesity, dyslipidemia, type 2 diabetes, and an elevated risk of ovarian cancer^1,2^.

Sarcopenia is a progressive skeletal muscle disorder involving the loss of muscle mass, strength, and function, with higher prevalence among older adults^3–5^. Its multifactorial etiology includes age-related changes in muscle protein synthesis, chronic low-grade inflammation, hormonal imbalances, and sedentary lifestyle. Emerging evidence suggests that sarcopenia is closely associated with metabolic disorders^3,4^. Clinically, sarcopenia is associated with adverse outcomes, including physical disability, increased risk of falls and fractures, reduced quality of life, and higher mortality rates^3,4^. Its diagnosis typically involves assessments of muscle mass, e.g., dual-energy x-ray absorptiometry (DXA), muscle strength (e.g., handgrip strength), and physical performance (e.g., gait speed)^6^. Given its significant disease burden, sarcopenia necessitates comprehensive strategies encompassing early diagnosis, nutritional interventions, resistance training, and pharmacological approaches to mitigate its progression and improve clinical outcomes^3,4,6^.

Despite emerging evidence of overlapping pathogenic mechanisms between endometriosis and sarcopenia, such as chronic inflammation and metabolic dysregulation, no prior study has directly investigated their association^2,3^. Given the high prevalence of both conditions and the current lack of clarity regarding their interplay, this study sought to examine the relationship between sarcopenia and endometriosis. In this study, we extracted data from The National Health and Nutrition Examination Survey (NHANES) 1999–2006 to investigate the incidence and risk factors for sarcopenia in women with endometriosis.

## Methods

### Study design and population

The National Health and Nutrition Examination Survey (NHANES) dataset is a nation-wide program to assess the health and nutritional status of adults and children in the United States. It was conducted by the National Center for Health Statistics (NCHS), which is a part of the Centers for Disease Control and Prevention (CDC). NHANES data cover a wide range of health-related items, including participants’ dietary intake, nutritional status, physical activity, medical conditions, and environmental exposures^3^.

**Fig. 1:**
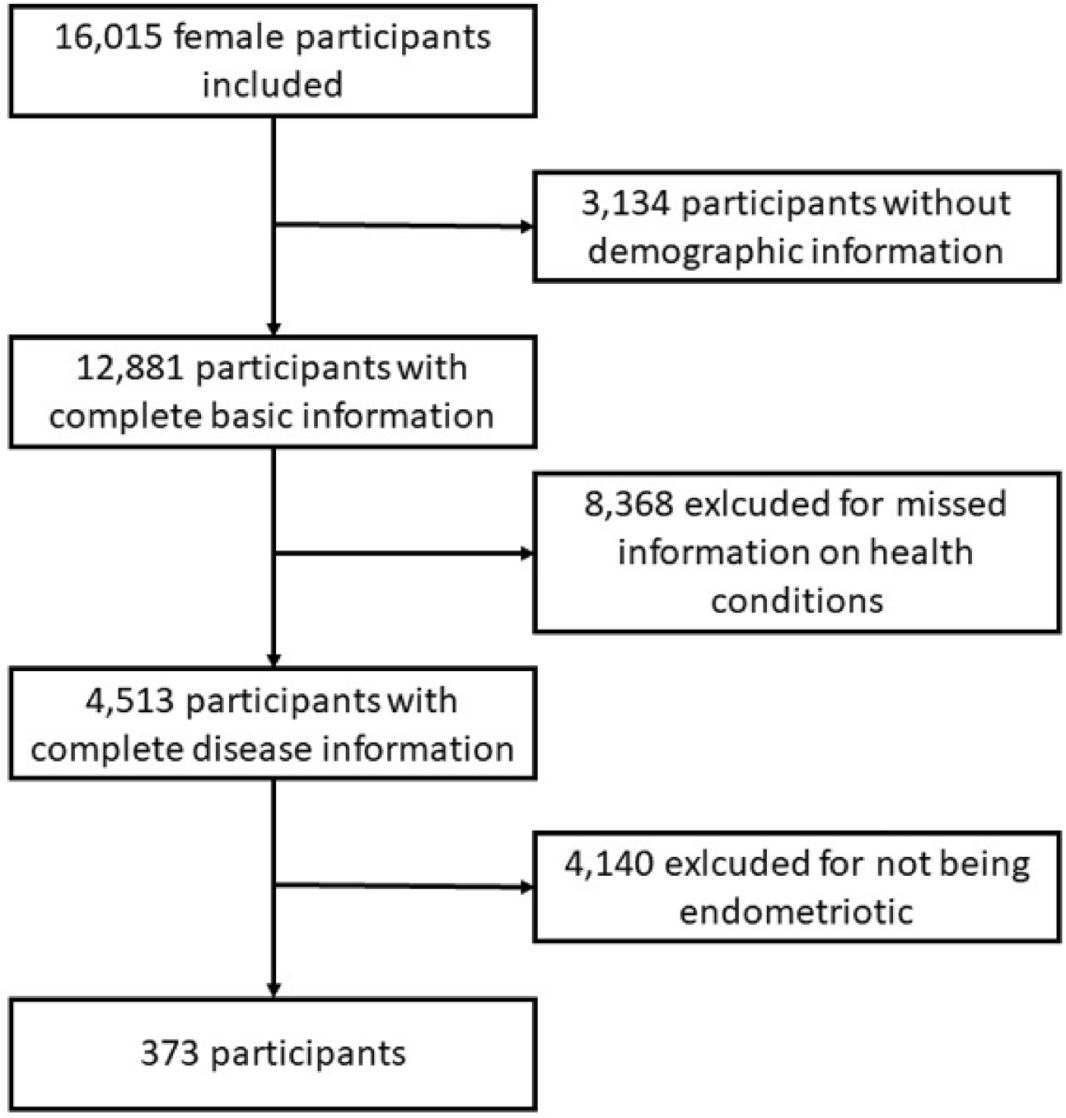
Flow chart to demonstrate the selection of participants in this study.

The cycles of 1999–2000, 2001–2002, 2003–2004, 2005–2006 were only cycles containing endometriotic status of participants in their reproductive health questionnaire (RHQ)^7^. Adult women aged 20 or older with self-reported information on endometriosis (RHQ_D, RHQ360, RHQ370) were included. Those with incomplete information on age, race, height, BMI, poverty income ratio (PIR), marital status, educational level, smoking habits, and a series of health conditions including hypertension, diabetes, cardiovascular diseases, asthma, emphysema or chronic bronchitis, cancer, or Dual-energy x-ray absorptiometry (DXA) data were excluded. As a result, 373 female participants made the finalist (Fig. 1).

### Definition of variables

Endometriosis was defined by a positive response to the question “Has a doctor or other health professional ever told you that you had endometriosis?” (RHQ360). Then they were asked about the age of the diagnosis (RHQ370). Sarcopenia was defined as appendicular skeletal muscle mass index (ASMI) ≤ 5.5. ASMI = ASM/height^2^ (m^2^, and appendicular skeletal muscle mass (ASM) is measured by accumulation of the lean mass of the arms and legs assessed by Dual-energy x-ray absorptiometry (DXA)^8,9^. The covariates were also collected, including age, race (1 = Mexican American, 2 = Other Hispanic, 3 = Non-Hispanic White, 4 = Non-Hispanic Black, 5 = Other Races), marital status (1 = married or living with partner, 2 = other status), educational level (1 = some college or associates degree, or above, 2 = high school or under), poverty income ratio (≥ 1.0 was defined as rich, while < 1.0 were defined as poor), smoking habits (1 = smoking was defined as smoked more than 20 times in life, SMQ150 between 1999 and 2004 or smoked at least 100 cigarettes in life, SMQ020 from 2005 to 2006. 2 = non-smoking)^10^. Physical activity was defined following the response to the physical activity questionnaire (PAQ), the average level of physical activity each day (PAQ180). The answer of 1 or 2 was defined as “1” in Table 1. This indicated mild physical activity, while the answer of 3 or 4 was defined as “2”, which suggestive of sufficient physical activities. Hormonal supplement was investigated via the reproductive health questionnaire (RHQ). Participants replied to the question of “Ever use female hormones”, and “1” indicated “Yes”, while “2” indicated “No”. (RHQ540). One or more self-reported conditions including hypertension, angina, heart attack, coronary diseases were defined as “Yes” to cardiovascular disease, while “No” if none was reported. Diabetes was also self-reported with “borderline” accounted as disease status. Pulmonary disease was defined with at least one of following disorders: asthma, emphysema or chronic bronchitis. Cancer status was defined as diagnosis of cancer, but the specific types were not further extracted. The duration of the disease course of endometriosis was calculated by participants’ age minus diagnostic age. Long disease duration was defined if the diagnosis was made more than five years ago.

**Table 1:** Baseline characteristics of endometriotic patients with or without sarcopenia.

Variables	Sarcopenia		*p*-value
	No (*N* = 325)	Yes (*N* = 48)	
Age (mean ± SD)	40.0 ± 8.5	40.3 ± 9.7	0.867
Race*			0.001
Mexican American	27 (8.3%)	3 (6.2%)	
Other hispanic	7 (2.2%)	0 (0%)	
Non-hispanic White	232 (71.4%)	25 (52.1%)	
Non-hispanic Black	50 (15.4%)	14 (29.2%)	
Other race	9 (2.8%)	6 (12.5%)	
BMI Classification (kg/m^2^)			0.483
Normal range (18.5–24.9)	197 (60.6%)	32 (66.7%)	
Underweight (< 18.5)	7 (2.2%)	0 (0%)	
Obese (≥ 30.0)	121 (37.2%)	16 (33.3%)	
Marital status			0.869
Married	214 (65.8%)	32 (66.7%)	
Single	111 (34.2%)	16 (33.3%)	
Educational level*			0.023
College or above	191 (58.8%)	37 (77.1%)	
High school or under	134 (41.2%)	11 (22.9%)	
Poverty to income ratio			0.559
Rich (≥ 1.0)	277 (85.2%)	43 (89.6%)	
Poor (< 1.0)	48 (14.8%)	5 (10.4%)	
Physical activity			1.000
Yes	261 (80.3%)	38 (79.2%)	
No	64 (19.7%)	10 (20.8%)	
Hormonal supplement			0.790
Yes	125 (38.5%)	20 (41.7%)	
No	199 (61.5%)	28 (58.3%)	
Smoking status			0.673
Yes	43 (13.2%)	8 (16.7%)	
No	282 (86.8%)	40 (83.3%)	
Cardiovascular status			0.257
Yes	90 (27.7%)	9 (18.8%)	
No	235 (72.3%)	39 (81.2%)	
Diabetic status			0.997
Yes	15 (4.6%)	2 (4.2%)	
No	310 (95.4%)	46 (95.8%)	
Pulmonary disease			0.899
Yes	48 (14.8%)	8 (16.7%)	
No	277 (85.2%)	40 (83.3%)	
Cancerous status			0.499
Yes	33 (10.2%)	7 (14.6%)	
No	292 (89.8%)	41 (85.4%)	
Duration of endometriosis^#*^	10.0 (5.0–17.0)	4.0 (3.0-11.5)	< 0.001
Longer than five years	247 (76%)	19 (39.6%)	< 0.001
Less than five years	78 (24%)	29 (60.4%)	

### Statistical analysis

All statistical analyses in this current study were conducted as per Centers for Disease Control and Prevention (CDC) guidelines. We adopted SPSS version 26.0 and an online database (www.mimicdb.com) to facilitate variable extraction and statistical analyses. Categorical variables were presented as percentages. Normally distributed continuous variables were presented as mean ± SD, while non-normal distribution was described as normally distributed variables are presented as the median (quartiles 1 and 3). Some continuous variables were transformed into ordinal variables. For example, the BMI was divided into “1” for participants within the normal range. Participants who were underweight were divided into “2”, while BMI ≥ 30 kg/m^2^ were “3”. The I^2^ test was adopted to compare the categorical variables, whereas the Mann-Whitney U-test was used for continuous parameters. We performed four models to further elucidate the correlation between the disease course and sarcopenia in endometriotic women. We then employed multivariate regression models without any adjustment to generate the crude model (Model 1). In Model 2, we adjusted for participants’ age, race and BMI. In Model 3, apart from covariates in Model 2, marital status, educational attainment, poverty income ratio (PIR), physical activity (PA), hormonal supplement status, and smoking status were adjusted. In Model 4, we adjusted for all covariates included in this study: age, race, BMI, MS, education, PIR, PA, hormonal supplement, smoking habits, cardiovascular disease, pulmonary disease, diabetes and cancer were adjusted.

## Results

### Baseline characteristics of included participants

The study enrolled 373 patients with endometriosis (Fig. 1). The average age of the endometriotic patients with and without sarcopenia were 40.3 and 40.0, respectively. Significantly higher risks of sarcopenia were seen in Non-Hispanic Black (29.2% Vs 15.4%) and other races (12.5% Vs 2.8%). Interestingly, patients with higher educational levels were disproportionally higher to be diagnosed with sarcopenia (77.1% Vs 58.8%). The body mass index (BMI) was not contrastingly different between sarcopenic and non-sarcopenic endometriosis patients. Smoking behavior and economic status were similar irrespective of sarcopenia. Additionally, health conditions were also insignificantly different. The detailed demographic information and healthy conditions were demonstrated in (Table 1).

### Univariate analysis

The univariate analysis in Table 2 evaluates several factors associated with sarcopenia, highlighting key demographic, socioeconomic, and the impact of health conditions influences. The analysis reveals that race plays a significant role, with individuals from racial minority showing a notably higher odds ratio (OR = 6.00, 95% CI 1.24–29.07, *p* = 0.026) for sarcopenia compared to Mexican Americans, while non-Hispanic Black individuals also exhibit a higher, though not statistically significant, risk (OR = 2.52, 95% CI 0.67–9.55, *p* = 0.174). BMI indicates that obese patients were associated with a slightly lower risk of sarcopenia (OR = 0.81, 95% CI 0.43–1.55, *p* = 0.530) compared to those with a normal BMI. While marital status does not appear to significantly affect the likelihood of sarcopenia, educational attainment is significantly associated with sarcopenia risk; individuals with a high school education or lower have a reduced odds ratio (OR = 0.42, 95% CI 0.21–0.86, *p* = 0.018) compared to those with college education or above. The data suggest that educational attainment may increase sarcopenic risks in endometriotic population. Other factors such as physical activities, hormonal use, smoking status, cardiovascular health, diabetes, pulmonary disease, and cancer status did not show significant associations with sarcopenia in this analysis. Furthermore, the duration of endometriosis diagnosis shows a profound impact, with those diagnosed for less than five years having a significantly higher odds ratio (OR = 4.83, 95% CI 2.57–9.09, *p* < 0.001) for sarcopenia compared to those diagnosed for more than five years, indicating that shorter duration of the endometriosis is associated with higher sarcopenia risk. These findings underscore the importance of racial background, educational attainment, and disease duration in understanding the risk factors for sarcopenia in endometriotic population.

**Table 2:** Univariate analysis of variables with sarcopenia.

Variables	OR (95% CI)	*p*-value
Race		
Mexican American	1.00 (reference)	
Other hispanic	0.00 (0.00-Inf)	0.987
Non-hispanic White	0.97 (0.27–3.43)	0.962
Non-hispanic Black	2.52 (0.67–9.55)	0.174
Other race	6.00 (1.24–29.07)	0.026
BMI Classification* (kg/m^2^)		
Normal range (18.5–24.9)	1.00 (reference)	
Underweight (< 18.5)	0.00 (0.00-Inf)	0.987
Obese (≥ 30.0)	0.81 (0.43–1.55)	0.530
Marital status		
Married	1.00 (reference)	
Single	0.96 (0.51–1.83)	0.911
Educational level*		
College or above	1.00 (reference)	
High school or lower	0.42 (0.21–0.86)	0.018
Poverty to income ratio		
Rich (≥ 1.0)	1.00 (reference)	
Poor (< 1.0)	0.67 (0.25–1.78)	0.423
Physical activity		
No	1.00 (reference)	
Yes	1.07 (0.51–2.27)	0.853
Hormonal supplement		
No	1.00 (reference)	
Yes	0.88 (0.47–1.62)	0.671
Smoking status		
No	1.00 (reference)	
Yes	1.31 (0.58–2.99)	0.519
Cardiovascular status		
No	1.00 (reference)	
Yes	0.60 (0.28–1.29)	0.194
Diabetic status		
No	1.00 (reference)	
Yes	0.90 (0.20–4.06)	0.889
Pulmonary disease		
No	1.00 (reference)	
Yes	1.15 (0.51–2.62)	0.731
Cancerous status		
No	1.00 (reference)	
Yes	1.51 (0.63–3.64)	0.357
Course of EMT		
Duration ≥ 5yrs	1.00 (reference)	
Duration < 5yrs	4.83 (2.57–9.09)	< 0.001

### Association between endometriosis and sarcopenia

We then assess the association between endometriosis and sarcopenia. The results were showed in (Table 3). In general, a shorter course of endometriosis (recent diagnosis less than five years) was consistent and strongly associated with sarcopenic risks across all models. The original odds ratio (OR) before any adjustment was 4.83, with a 95% CI of 2.57 to 9.09, indicating a positive correlation. After adjusting for basic covariates such as age, race, BMI, Model 2 still revealed positive association with an OR at 4.61 (95% CI: 2.40–8.86). Furthermore, the association is particularly pronounced after adjusted for age, race, BMI, marital status, educational level, poverty income ratio, physical activity, hormonal use and smoking habits (OR: 5.65, 95% CI 2.84–11.25). After adjusted all covariates, the positivity remained with an OR at 5.61 (95% CI 2.80-11.25).

**Table 3:** Association between the course of endometriosis and sarcopenia.

	Crude model	Model 2	Model 3	Model 4
Total OR	4.83 (2.57–9.09) *p*-value < 0.01	4.61 (2.40, 8.86) *p*-value < 0.01	5.65 (2.84, 11.25) *p*-value < 0.01	5.61 (2.80-11.25) *p*-value < 0.01

Model 1: crude model.

Model 2: adjust for age, race, BMI.

Model 3: adjust for age, race, BMI, marital status, education, poverty income ratio, physical activity, hormonal supplement status, and smoking status.

Model 4: adjust for age, race, BMI, marital status, education, poverty income ratio, physical activity, hormonal supplement status, smoking; disease status of diabetes, cardiovascular diseases, pulmonary diseases, and cancer.

## Discussion

The study involved 373 participants from NHANES dataset 1999–2006 to investigate the link between endometriosis and sarcopenia. The average age of patients with and without sarcopenia was both around 40 years. Notably, racial background and educational level emerged as significant factors; racial minorities and individuals with higher educational attainment faced higher sarcopenia risks. In contrast, BMI, marital status, physical activities, hormonal supplement use, smoking behavior, and economic status were not significantly associated with sarcopenia risk in endometriotic population in this study. Furthermore, patients diagnosed with endometriosis for less than five years demonstrated a much higher risk of developing sarcopenia, an association that remained robust even after adjusting for various covariates. These results underscore the critical influence of race, education, and disease duration on the risk of sarcopenia in endometriotic patients. These factors should be carefully considered in future clinical practice.

By far, no study has reported the correlation between endometriosis and sarcopenia, but there may be a theoretical overlap in pathogenesis between the two disorders^3,4,6^. Theoretically, the shared chronic inflammation can be one of the underlying mechanisms^11,12^. Endometriosis is characterized by chronic inflammation, demonstrated by elevated pro-inflammatory cytokines, such as TNF-α, interleukin-1 (IL-2), and interleukin-6 (IL-6)^13,14^. These cytokines can degrade and inhibit muscle protein synthesis, paving the way to developing sarcopenia. The cytokines’ pro-inflammatory effect further stimulates reactive oxygen species (ROS), perpetuating oxidative stress, which plays a significant role in the development and progression of endometriosis and sarcopenia^15,16^. Hormonal imbalance can be another causal factor for concurrent endometriosis and sarcopenia. Abnormally increased estrogen may induce the systemic production of ROS through its metabolism. The production in turn, could cause sarcopenia in some patients^17,18^. Lifestyle factors may also add to the shared pathogenesis as endometriosis could cause chronic pelvic pain and reduced physical activity^5^. Long-term inactive lifestyle predisposes individuals to muscle weakness and atrophy, which is a known risk factor for sarcopenia^5^. However, this may not explain our findings that endometriotic patients were similar in physical activities regardless of sarcopenia status. The study revealed unexpected results that a shorter course of endometriosis (diagnosis made in less than five years) is positively associated with sarcopenia. This may be explained by several possible hypotheses. One of them suggests a shorter duration may be owing to delayed diagnosis of EMT. As the presentation of endometriosis is similar to many other pelvic disorders, such as irritable bowel syndrome (IBS), chronic appendicitis, pelvic inflammatory disease (PID), fibroids, the diagnosis is often delayed^1^. The occult endometriosis without medical interventions could predispose patients to long-term complications including sarcopenia. From another perspective, a shorter course of later onset endometriosis may imply a disease rapid progression. Although not malignant in nature, endometriotic cells’ biological behavior echoes tumor progression^1,11^. The rapid disease progression may add more sarcopenic risks to individuals. Additionally, racial and educational backgrounds also played a contributing role. Further sub-group analysis across different racial contexts may help explain the notable diversity observed.

This study has several strengths. Firstly, it is the first research article to report correlation between endometriosis and sarcopenia. The correlation may guide healthcare providers in their daily practice managing endometriotic patients with or without sarcopenia. In time diagnosis and management may reduce disease burden and physical and psychological suffering. Secondly, the correlation between the two health conditions can be extrapolated outside the U.S. As the data were extracted from NHANES, the diverse racial backgrounds enabled reasonable representativeness while extrapolating the conclusion. Thirdly, we proposed four models to demonstrate step-by-step the positive correlation between shorter duration of endometriosis and sarcopenic risks. This association was independent of demographical, lifestyle and other common non-communicable chronic diseases. However, there are some limitations to this study. First, the sample size is small, with only less than 400 participants included. This could introduce bias, and we expect future research to expand the sample size to better elucidate the association between the two health conditions. Second, racial differences were significant in developing sarcopenia, and was most remarkable in Other Races. Studies with further divided racial, ethnical backgrounds may better guide the medical practice in the future. Third, the endometriosis was self-reported, it could lead to result bias, and deviated association when compared with risks of developing sarcopenia. This was prevalent in NHANES studies as a range of disorders were self-reported instead of diagnosed by laboratory or imaging methods.

## Conclusion

In conclusion, this study suggested that higher educational degree and shorter course of endometriosis were positively associated with a higher risk of sarcopenia. Future prospective cohort study and research experiments may further elucidate the relationship.

## Data Availability

The datasets used in the manuscript are publicly available, which can be accessed at https://www.cdc.gov/Nchs/Nhanes/.
